# Obturator hernia: a delayed diagnosis. A case report with literature review

**DOI:** 10.1093/jscr/rjaa599

**Published:** 2021-01-25

**Authors:** Zohaib Siddiqui, Mohammed Khalil, Aoff Khalil, Shoaib Saeed

**Affiliations:** General Surgery, Maidstone & Tunbridge Wells NHS Trust, Tunbridge Wells, UK; General Surgery, Maidstone & Tunbridge Wells NHS Trust, Tunbridge Wells, UK; General Surgery, Maidstone & Tunbridge Wells NHS Trust, Tunbridge Wells, UK; General Surgery, Medway NHS Trusty, Medway, UK

**Keywords:** hernia, general surgery, emergency surgery, obturator hernia

## Abstract

Obturator hernias are classically difficult to diagnose, have a high mortality and are an uncommon cause of intestinal obstruction. They are usually found in thin, elderly female patients. We present a case of a misdiagnosed 89-year-old female who presented to accident and emergency with a short history of abdominal pain. The diagnosis of an incarcerated obturator hernia was confirmed after re-discussion of computed tomography scan with the consultant radiologist in the morning. The patient underwent emergency laparotomy and the defect reduced. The patient recovered well post-operation; however; on the fourth day post-operation, the patient suffered a cardiac arrest. We report this case as a reminder to our health care colleagues to be mindful of elderly female patients who present with small bowel obstruction due to the high risk of mortality of this type of hernia.

## INTRODUCTION

Obturator hernias are rare and difficult to clinically diagnose [1]. Medical literature reports the incidence of obturator hernias to be between 0.05 and 2.2% of all hernias [1]. They are often described as ‘little old lady’s hernia’, as they typically affect elderly emaciated female patients [2]. The diagnostic challenge is due to the non-specific nature of the presentation [3]. The mortality rate for this type of hernia has been reported as 12–70%, and the presentation tends to be acute [2, 3]. A more recent study in 2013 found a mortality of 48% [4]. A differential diagnosis of an obturator hernia should be present with any thin, elderly female patient with signs or symptoms of obstruction.

## CASE PRESENTATION

An 89-year-old female presented to the emergency department with a short history of severe lower abdominal pain which radiated to her back. The pain was described as a sharp intermittent pain. She had vomited once (non-bilious, nil blood). She had not opened her bowels for 24 h. She had no fevers or dysuria.

She had a medical history of an abdominal aortic aneurysm (AAA), chronic obstructive pulmonary disease (COPD) and rheumatoid arthritis.

On examination, she had a soft abdomen but was tender in the left iliac fossa with no guarding. She had no palpable masses. Glasgow coma score 15/15.

**Figure 1:**
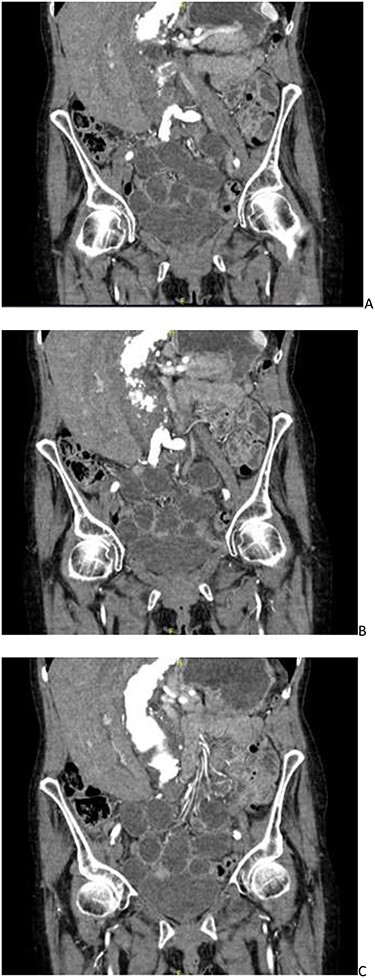
CT angiogram showing a left-sided obturator hernia; (**A**–**C**) progressing posteriorly in a coronal plane.

Her observations on admission were: respiratory rate: 16, saturations: 93%, blood pressure: 117/64, heart rate: 75, alert and temperature: 36.8 Celsius. Her pain score was recorded as 9/10.

Her blood tests were unremarkable and no signs of infection noted.

She was suspected of having a worsening AAA, and thus a computed tomography (CT) angiogram was organized. Overnight this was reported as a stable AAA without rupture with incomplete small bowel obstruction, possibly due to faecal loading of the caecum. The patient was then treated as such with laxatives and an enema. The patient did not improve, and clinically, her symptoms did not correlate with the CT findings. The CT report was re-discussed the following day and an addendum was made. The CT scan was now reported as a mechanical small bowel obstruction secondary to a left obturator hernia, which contained a small segment of ileum as seen in Fig. 1.

**Figure 2:**
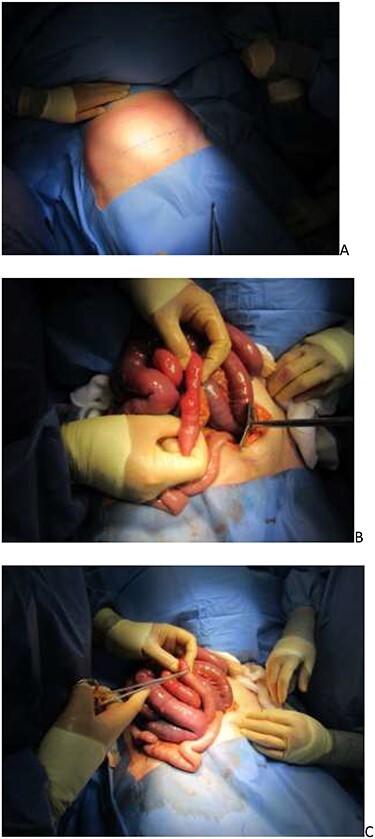
(**A**) Pfannenstiel incision marked; (**B**) incarcerated ileum and (**C**) serosal tear repaired.

The patient was treated as an small bowel obstruction (SBO) and an NG tube was inserted and aspirated, and she was kept nil by mouth and was given intravenous fluids. Once the diagnosis was confirmed by CT, the patient was prepped for emergency surgery on the CEPOD list. A regional spinal block was used to reduce the anaesthetic complications of intubation. The laparotomy was performed using a Pfannenstiel incision as seen in Fig. 2. Dilated small bowel loops were seen and a 3-cm incarcerated loop of ileum with a serosal tear was reduced from the obturator canal. There were no signs of bowel necrosis and no objective obturator defect was seen or felt. The patient recovered well in the ITU and was stepped down to the wards. On her fourth day post-op, unfortunately, she suffered a cardiac arrest and passed away.

## DISCUSSION

An obturator hernia is characterized by a herniation of bowel or abdominal content between the obturator and pectineus muscles into the obturator canal [5]. This type of hernia has been reported as being up to nine times more common in women versus men [5]. This could be due to their wider pelvis, larger diameter and more triangular shape of the obturator canal [5]. In general, it is thought to result from the progressive weakening of pelvic floor muscles, which may be attributed to age, multiparity or emaciation [6]. Most commonly, these hernias are seen in elderly, female patients who are emaciated. A loss in protective preperitoneal fat from malnutrition can increase the area around the obturator canal, allowing herniation [6]. Risk factors such as COPD, constipation and ascites have been noted to contribute to the development of obturator hernias due to increased intraabdominal pressures [6].

Diagnosis of obturator hernias preoperatively is challenging [5]. In our case, the diagnosis of faecal impaction leading to small bowel obstruction was incorrect. The most common clinical test mentioned in the literature is the Howship–Romberg sign, which is when pain is elicited along the distribution of the obturator nerve due to compression of the nerve [7]. This only presents in 15–50% of patients with obturator hernias [7]. However, the Hannington–Kiff sign is more specific but a less well-known sign. This typically presents with signs of intestinal obstruction, such as vomiting, constipation, nausea and abdominal pain. Obturator hernias are said to be the cause of up to 1.6% of mechanical small bowel obstruction [8]. CT scan with contrast is the modality of choice in an emergency setting; however, even then, it can be missed, leading to delays in surgery [9]. Delays in surgery are one of the key factors affecting bowel resection. In one recent study, three patients had a CT scan and the obturator hernia was missed initially, leading to a delay in diagnosis and thus surgery [9]. Similarly, in the case we report, the initial CT report missed the subtle obturator hernia leading to a delay in surgery.

Laparoscopic surgery is becoming more popular when repairing obturator hernias and even cases of two-staged approaches are mentioned in literature [10]. This is due to the reduced post-operative recovery times due to fewer post-op complications, such as ileus, respiratory complications and pain [10]. A mesh or suture repair is performed if a defect is found to prevent reoccurrence [4]. The benefit of the mesh repair has generally outweighed the risk of infection, and a review of obturator hernia repair found a 0% reoccurrence rate for mesh repairs over 3 years [10].

## CONCLUSION

We report this rare case as a reminder to health care colleagues working in primary and secondary care to acknowledge the importance of obturator hernias and appreciate the difficulties in diagnosis. CT scans remain the gold standard [9] and should be reviewed thoroughly, and if the diagnosis of obturator hernia is suspected, it should be mentioned on the scan request. Obturator hernias account for nearly 2% of mechanical small bowel obstruction and their mortality rate has been reported as nearly 50% in recent studies [4], making these one of the most lethal hernias reported in literature.

## CONFLICT OF INTEREST STATEMENT

None declared.

## FUNDING

None.
