# Corrigendum to: Obturator hernia: a delayed diagnosis. A case report with literature review

**DOI:** 10.1093/jscr/rjab238

**Published:** 2021-05-18

**Authors:** Zohaib Siddiqui, Mohammed Khalil, Aoff Khalil, Shoaib Saeed

**Affiliations:** 1 General Surgery, Maidstone & Tunbridge Wells NHS Trust, Tunbridge Wells, UK; 2 General Surgery, Medway NHS Trusty, Medway, UK

In the originally published version of this manuscript, there was a spelling error in the last author's forename. The correct spelling is: Shoaib Saeed. This error has been corrected online.

